# Protrichocysts: a hybrid defense extrusive organelle bridging mechanical projection and chemical secretion in ciliates

**DOI:** 10.1016/j.crmicr.2025.100539

**Published:** 2025-12-23

**Authors:** Kangqiao Dong, Peilin Cai, Liping Lyu, Juan Yang, Yi Wu, Letizia Modeo, Xiao Chen, Jing Xu, Xinpeng Fan

**Affiliations:** a School of Life Sciences, East China Normal University, Shanghai 200214, China; b Key Laboratory of Evolution & Marine Biodiversity (Ministry of Education), and Institute of Evolution & Marine Biodiversity, Ocean University of China, Qingdao, 266003, China; c Marine College, Shandong University, Weihai 264209, China; d Department of Biology, University of Pisa, Pisa, 56126, Italy; e Centro Interdipartimentale di Microscopia Elettronica (CIME), University of Pisa, Pisa, 56126, Italy; f Center for Instrument Sharing (CISUP), University of Pisa, Pisa, 56126, Italy; g Key Laboratory of Chemical Biology and Molecular Engineering of Ministry of Education, School of Life Sciences, Shanxi University, Taiyuan, 030006, China; h Institute of Advanced Agricultural Science and Technology, East China Normal University, Shanghai, 200241, China

**Keywords:** Electron microscopy, Exosomes, Extrusomes, Function, HPLC-MS/MS, *Pseudourostyla cristata*, Ciliophora

## Abstract

Extrusive organelles (extrusomes) represent a specialized class of dense-core granules in protists that exhibit remarkably complex morphology and secretory mechanisms compared to their metazoan counterparts, reflecting key adaptations in single-celled eukaryote evolution. Protrichocysts, which are among the most intricate projectile extrusomes in ciliates, have thus far been understood primarily in terms of their general morphology. In this study, we employed a comprehensive approach to investigate the protrichocysts of *Pseudourostyla cristata*, thereby expanding our understanding of these organelles. Through predator-prey interaction experiments and electron microscopy, we demonstrated the ejection of protrichocysts in response to a predator’s attack and revealed that their ejection process consists of three main stages: (i) cap dissolution, (ii) dual-phase body expansion, and (iii) controlled shaft exposure. Histochemical analysis indicated that extruded protrichocysts contain acid mucopolysaccharides and microtubules. SDS-PAGE and HPLC-MS/MS analyses identified the major protein bands and the presence of glycoproteins, phosphoproteins, and potential defense-related proteins. Single-cell transcriptome analysis suggested the involvement of conserved carboxypeptidase-mediated extrusome biogenesis. These findings establish protrichocysts as multifunctional organelles that employ a unique hybrid defense strategy combining mechanical projection with chemical secretion. Their distinctive microtubular cap architecture and specialized matrix composition further suggest potential roles in intercellular communication. Additionally, this research improves our understanding of protist extrusome biology and provides new insights into the evolution of eukaryotic secretory systems.

## Highlights

• Protrichocysts launches a millisecond-scale, three-stage ejection that unites trichocyst-like mechanical ejection with toxicyst-like chemical release, providing ciliates an inducible hybrid defense against predators.
• First comprehensive histochemical and proteomic map of its protrichocysts uncovers acid mucopolysaccharides, microtubules and candidate toxic proteins, implying roles beyond defense such as intercellular signaling.
• Protrichocysts fully regenerate within 12 h after experimental ablation, driven by a transcriptional burst of ribosomal and carboxypeptidase genes that mirror metazoan dense-core-granule biogenesis.
• Comparative multi-omics reveal conserved molecular architectures between these ciliate extrusomes and metazoan exosomes/dense-core granules, highlighting a unified eukaryotic secretory heritage.

## Graphical abstract

**Figure:**
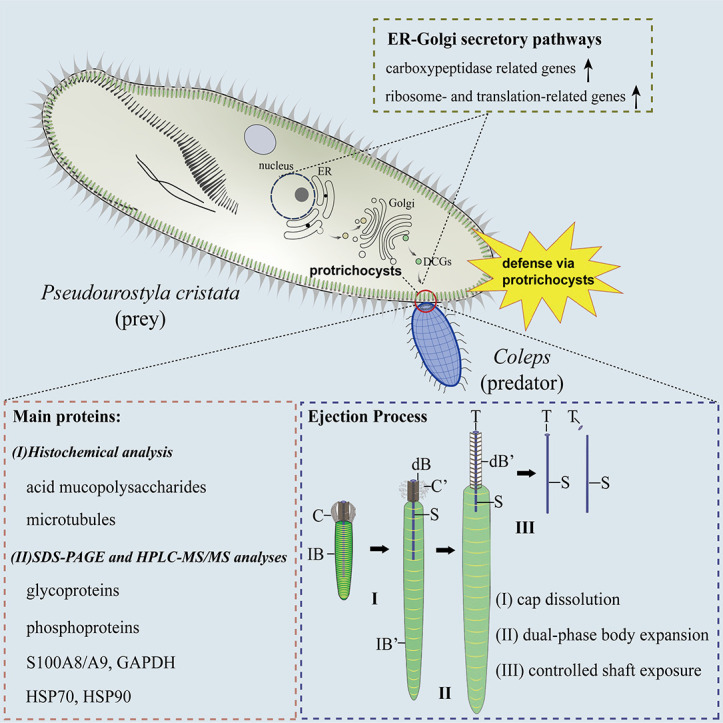

## Introduction

Dense core granules (DCGs) are regulated secretory vesicles characterized by their electron-opaque cores and larger size (approximately 60–300 nm in diameter) compared to synaptic vesicles (Meldolesi et al., 2004). The condensed core of DCGs may comprise peptide hormones, various ions, small molecules, monoamine neurotransmitters and other proteins, and serve an equally wide array of functions in regulating and maintaining various physiological functions of eukaryotes, like ingestion, reproduction, ion balancing, individual growth and development, etc. (Thureson-Klein and Klein, 1990; Walch-Solimena et al., 1993).

In single-celled eukaryotes, DCG-like organelles are usually included in the so-called “extrusive organelles” or “extrusomes” (Elde et al., 2007; Turkewitz, 2004; Vayssié et al., 2000). These are ejectable membrane-limited organelles inserted in the cortex that can release their contents to the extracellular space by fusing their membrane with the plasma membrane in response to various stimuli; cells do not change their morphology during extrusion of extrusomes and can reform new extrusomes in several hours or days (Buonanno and Ortenzi, 2016; Froissard et al., 2004; Harumoto and Miyake, 1991; Hausmann, 1978; Rosati and Modeo, 2003). With over 15 identified types, extrusome morphology often provides clues to phylogenetic relationships. For instance, projectile extrusomes have a more limited distribution and can be a synapomorphy for distinct lineages within Ciliophora (Rosati and Modeo, 2003). Their functions are diverse, including roles in defense, predation, cyst wall formation, photosensitivity, and extracellular communication (Hausmann, 1978; Kugrens et al., 1994; Rosati and Modeo, 2003). Therefore, the study of extrusomes is significant for understanding both protist adaptations and eukaryotic evolutionary relationships from a cellular perspective (Elde et al., 2007; Rosati and Modeo, 2003).

Protrichocysts, named for their similarity to the spindle trichocysts of *Paramecium*, are found in the hypotrichous family Pseudourostylidae (Fan et al., 2025; Jerka-Dziadosz, 1970). Protrichocysts exhibit unparalleled complexity, ejecting an enokitake-like structure under fixative stimulation (Buonanno and Ortenzi, 2016; Suganuma, 1973; Tokuyasu and Scherbaum, 1965; Zhang et al., 2007) and being degraded via autophagy during encystment (Grim and Manganaro, 1985; Zhang et al., 2011). Despite their intriguing features, fundamental cellular investigations into protrichocysts remain conspicuously lacking. As a result, the biogenesis, ejection dynamics, biochemical composition, and functional roles of protrichocysts are entirely uncharacterized. These knowledge gaps severely impede the understanding of extrusome biology and their evolutionary significance in ciliate evolution.

To address these critical gaps, this study was carried out on *Pseudourostyla cristata* and employed an integrative approach to: (1) elucidate the detailed ejecting process and the reaction of protrichocysts during a predator-prey interaction experiment using electron microscopy (TEM and SEM); (2) define the molecular composition via histochemistry and proteomics (SDS-PAGE/HPLC-MS/MS); (3) reveal the regeneration dynamics and the key transcriptional regulation pathways via single-cell transcriptome; and (4) evaluate evolutionary parallels with extrusomes of ciliates and other eukaryotic secretory systems through transcriptomic and functional comparisons.

## Materials and methods

### Cell culturing and experimental protrichocysts ejection by means of a predator-prey interaction

*Pseudourostyla cristata* cells were cultured in autoclaved lake water and fed on *Chlamydomonas reinhardtii*. The possible ejection of protrichocysts in response to predators’ attack was verified by means of an experimental predator-prey (i.e., *Coleps-P. cristata*) interaction (Buonanno et al., 2005). In 250 μL clean water, we mixed 50 starved *Coleps* with 10 *P. cristata* cells constituting an experimental group; three sets of parallel experiments were performed, each of them lasting five minutes. Each predator-attacked prey was quickly removed from the water and treated for SEM (see below for details); all these cells were examined in parallel to 10 non-exposed-to-predator *P. cristata* cells (control group) processed under identical experimental and SEM conditions.

### Electron microscopy

For the examination of protrichocysts’ ejection induced by a predator-prey interaction, *P. cristata* cells were treated for scanning electron microscope (SEM) mainly according to the procedure reported by Gu and Ni (1993). Cells were chemically preserved in a mixture of 2 % OsO_4_ and saturated HgCl_2_ in a volume ratio of 1:6 for 10 min (quick fixation, thus no fixative-caused extrusion). Observation was performed using a Hitachi S-4800 SEM at an accelerating voltage of 3 kV.

SEM observation was also applied to study the discharging processes of protrichocysts after chemical stimuli treatments (see below for details). In this case, treated cells were let adhere onto polylysine coated slides first and then placed in a fixative composed of 2 % OsO_4_ and saturated HgCl_2_ in a volume ratio of 2:7 to let the protrichocysts eject.

For the observation of protrichocysts ultrastructure at the transmission electron microscope (TEM) samples were prepared following Gu and Ji (1996), sectioned using a Leica EM UC7 ultramicrotome, stained with uranyl acetate/lead citrate, and examined using a Hitachi HT7700 TEM at 80 kV.

### Chemical stimuli to experimentally induce the ejection of protrichocysts and histochemical analyses

Three solutions known to trigger the ejection of extrusomes were tested on *P. cristata* cells under a stereomicroscope. These were: (1) a solution of 11 mg/mL calcium chloride and 0.0025 % alcian blue 8GS (Solarbio, China); calcium chloride can induce the discharging and the alcian blue concurrently stains protrichocysts allowing to perform an additional histochemical analysis (Kumar et al., 2015; Tiedtke, 1976); (2) a 30 mg/mL methyl green-pyronin solution (Sangon Biotech, China) (Foissner, 1991), which induces the most violent ejection (i.e., with the maximal ejection distance and a full morphological extension of the extrusome) and therefore allows further SEM observation of morphological details during the ejecting process of protrichocysts; (3) a 0.1 mg/mL acridine orange (AO) solution (Sangon Biotech, China) (Lumpert et al., 1992), which keeps the best integrity of the ejected contents and therefore was used for further protrichocyst protein acquisition and HPLC-MS/MS (see below); AO concurrently stains the protrichocysts, allowing to perform an additional histochemical analysis.

In each of these three different treatments, 20 µL stimulating chemical solution was mixed with *P. cristata* cells fluid containing 50 cells in a volume ratio of 1:1. Ejection happened within a second and staining was achieved within a minute. FLUTAX-II (Glpbio, USA) labeling and fluorescence microscope observation was applied to test the presence of a microtubule component in the protrichocysts (Wang et al., 2024).

### Protrichocyst protein acquisition and SDS-PAGE analysis

*Pseudourostyla cristata* cells were cultured in 20 Petri dishes (12 cm diameter) with *C. reinhardtii* as food reaching a concentration of approximately 10⁶ cells/L. Then cells were starved (for at least 48 h) and sequentially filtered (filter pore size: 70 μm), centrifuged (650 rpm, 5 min), and manually purified under a stereoscope to be suitable for the protrichocyst ejection induction treatment with 0.1 mg/mL AO. Secreted protrichocysts were collected from the bottom of the Petri dishes by washing away cells, then dissolved in 2.5 % RIPA lysis buffer. After centrifugation (14,000 rpm, 10 min, 4 °C), pellets were lyophilized and resuspended in ultrapure water. Proteins >30 kDa were concentrated using ultrafiltration (Millipore), with concentration determined by BCA assay (10 µg/µL). SDS-PAGE analysis was performed by denaturing samples in Laemmli buffer at 100 °C for 3 min followed by electrophoresis (80 V on 5 % stacking gel and 120 V on 10 % resolving gel), with a protein visualization by means of Coomassie blue/silver staining and Odyssey CLX imaging.

### Identification of proteins based on high performance liquid chromatography-electrospray tandem mass spectrometry (HPLC-MS/MS)

The target protein band was excised from SDS-PAGE, de-stained, and subjected to in-gel digestion. Proteins were reduced by 10 mM dithiothreitol/ 100 mM NH_4_HCO_3_ for 30 min at 56 °C, alkylated with 200 mM lodoacetamide/100 mM NH_4_HCO_3_ in the dark at room temperature for 30 min, briefly rinsed in 100 % acetonitrile (ACN), and then digested overnight in 25 mM NH_4_HCO_3_ with 12.5 ng/μl trypsin. Peptides were extracted with 60 % acetonitrile/0.1 % trifluoroacetic acid, lyophilized, and resuspended in 0.1 % formic acid (FA) for HPLC−MS/MS analysis.

Liquid chromatography separation was performed on a C18 reversed-phase column (75 μm × 10 cm) using a linear gradient of 5–100 % buffer B (84 % ACN/0.1 % FA) at a flow rate of 300 nL/min. Mass spectrometry analysis was conducted on a Q-Exactive mass spectrometer (Thermo Fisher Scientific) in positive ion mode, using a data-dependent top-20 method with a survey scan resolution of 70,000 and higher-energy collisional dissociation fragmentation at 27 eV.

MS/MS spectra were analyzed using MaxQuant 1.6.14. Proteins were identified by searching against two databases: *P. cristata* transcriptome (Pan et al., 2020) (NCBI: SRR10854670), *P. cristata* genome (Jin et al., 2024). Additional parameters were as follows: 20 ppm peptide mass tolerance; 0.1 Da MS/MS tolerance; tryptic digestion with ≤2 missed cleavages; fixed carbamidomethylation (C); variable modifications including oxidation (M), glycosylation (+180.16 Da), and phosphorylation (+79.97 Da). Functional annotation of proteins was conducted with Blast2GO (v 6.0). For each Gene Ontology (GO) category—Biological Process (BP), Cellular Component (CC), and Molecular Function (MF)—the top ten terms were sorted based on unique peptide counts and visualized using GraphPad Prism (v10.5.0). Theoretical isoelectric points were calculated via ExPASy ProtParam. Sequence similarity clustering (cd-hit v4.8.1, 95 % threshold) compared *P. cristata* transcriptomic and genomic datasets (Puglia et al., 2020; Wenger and Galliot, 2013).

### Low-input transcriptomic analysis of protrichocyst regeneration

*Pseudourostyla cristata* cells were first treated with 0.1 mg/mL AO to remove protrichocysts, then washed in culturing water and cultured normally to allow for extrusomes regeneration. Regeneration was monitored by protargol staining (Wilbert, 1975), and the time-point at which approximately 50 % of the protrichocysts had re-formed was chosen as the regeneration stage (RS) for transcriptomic analysis. For RNA extraction, five to seven cells from the RS and from control vegetative-stage (VS) were collected in three biological replicates, lysed, and immediately flash-frozen. Low-input RNA extraction (SMARTer® Ultra™ Kit, USA) was followed by cDNA synthesis (Oligo dT priming) and library preparation (TruePrep DNA Library Prep Kit, China, transposon-based fragmentation). Single-stranded circularized libraries were amplified via rolling circle amplification into DNA nanoballs for PE100/150 sequencing on BGI platforms.

Transcriptome assembly followed the protocol described by (Yang et al., 2023). The unigenes were annotated by blasting against the following databases: Kyoto Encyclopedia of Genes and Genomes (KEGG), GO, EuKaryotic Orthologous Groups (KOG), Nucleotide database (Nt) and Non-redundant database (Nr) of NCBI, SwissProt, and Pfam. Read counts of each gene were calculated by RSEM v1.2.8 (Li and Dewey, 2011). DEseq2 (v1.36.0) was used to perform the normalization and comparison of gene expression levels during regeneration stage (RS) and vegetative stage (VS) (Love et al., 2014). Differentially expressed genes (DEGs) were quantified using the FPKM (Fragments Per Kilobase of transcript per Million reads) method (Mortazavi et al., 2008). DEGs were defined as those with a |log2(Fold Change)| > 1 and adjusted q-value ≤ 0.05). Principal component analysis (PCA) was carried out using the princomp function of R package 'ggplot2′ v3.4.1 (Ginestet, 2011). Functional and pathway enrichment of DEGs based on GO (Harris et al., 2004) and KEGG (Kanehisa et al., 2023) were carried out using the enricher function of R package 'clusterProfiler' with q-value < 0.1 (Yu et al., 2012), identified carboxypeptidase-related genes in GO enrichment, visualized via heatmaps using TBtools v2.3.15 (Chen et al., 2020).

## Results

### General ultrastructural morphology of protrichocysts

**Fig. 1:**
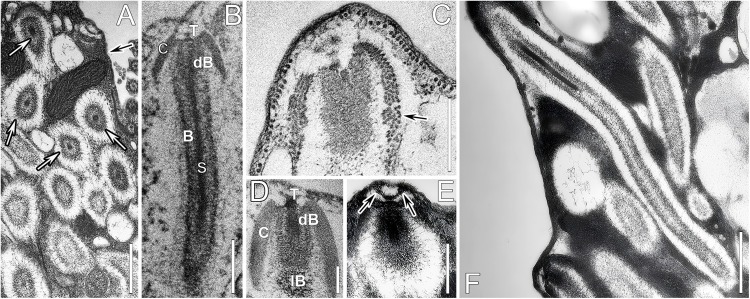
Transmission electron micrographs of protrichocysts of *Pseudourostyla cristata*. **(A)** Protrichocysts (white arrows) are abundantly distributed under the pellicle (black arrow). **(B, D)** Longitudinal thin sections of resting protrichocysts, showing the four parts, i.e., a cap (C), a body (B), a nail-head shaped tip (T), and a central shaft (S); the body can be further divided into the dense anterior part (dB) and less dense posterior part (lB). **(C)** The cap consists of regularly arranged microtubules (black arrow). **(E)** The margin of the tip seems to be in contact with a docking site of protrichocysts in the pellicle (black arrows). **(F)** Protrichocyst ejected within the cell. Scale Bars = 1 μm (**A, F**), 0.5 μm (**B, C, E**), 0.2 μm (**D**).

Protrichocysts were approximately 2–3 μm in length and 0.5–0.8 μm in diameter, comprising a tip, a cap, a central shaft, and a body that surrounded the shaft (Fig. 1A–D). The cap was approximately 0.8–1 μm in diameter and consisted of approximately 4–5 layers of microtubules (Fig. 1C/D). A nail-head shaped tip was in the hollow cavity of the cap, and the margin of the tip likely facilitates the docking of the protrichocyst to the pellicle (Fig. 1E). The central shaft measured 1.5 μm long and 90–100 nm in diameter. The body wrapping the shaft, was composed of a dense anterior part (dB) (about 0.4 μm in length and 0.2 μm in diameter) and a less dense posterior part (lB) (about 2 μm long and 0.4 μm in diameter) (Fig. 1D). Protrichocysts with a notably elongated lB, measuring roughly three times the length of resting-state extrusomes, were occasionally observed within the cell (Fig. 1F).

### Protrichocysts ejection after predator-prey interaction

**Fig. 2:**
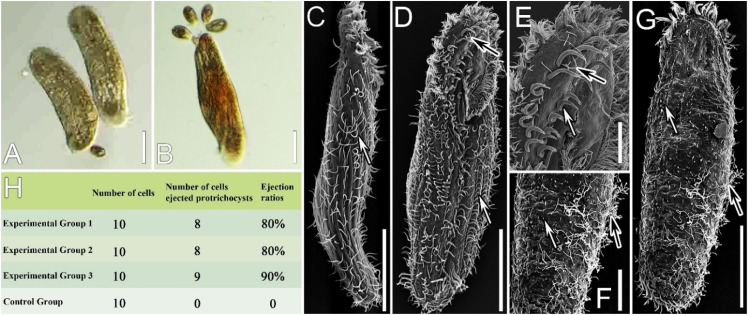
*Coleps*-*Pseudourostyla cristata* (predator-prey) interaction and the ejection of the protrichocysts in control (i.e., preys not exposed to predators) and experimental (i.e., preys exposed to predators) groups. **(A, B)** Prey-predator interaction at the light microscope: two *P. cristata* cells, the one on the left is attacked in its anterior region by a *Coleps* cell **(A)**; and four *Coleps* cells attacking another *P. cristata* cell in its posterior region **(B). (C‒G)** Scanning electron micrographs of cells of the control (**C**) and the experimental groups (**D‒G**) respectively. Protrichocysts ejection was not observed in the control group cells (**C**), while observed in the anterior portion of the ventral surface (**D, E**) and the posterior portions of the dorsal surface (**F, G**) of experimental group cells. Black arrows indicate the ejected protrichocysts, white arrows indicate cirri or cilia. **(H)** Recap table of the number of cells that ejected protrichocysts and ejection ratios in the three predator-prey interaction experimental replicates. Scale bars = 50 μm (**A, B, C, D, G**), 20 μm (**E, F**).

After mixing the predator *Coleps* sp. with the prey *P. cristata*, their interactions became effective in 1 min. Under the stereomicroscope, the interaction process between the two organisms was recorded (Fig. 2A, B; Supplementary Fig. S1A–D). *Coleps* attacked *P. cristata* with its anterior part (Fig. 2A, B), where its buccal field, with the inserted toxicysts, is located. This contact likely involved the ejection of the toxicysts by *Coleps*, which have been proved to be primarily involved in immobilizing and capturing the prey thanks to their toxic content (Buonanno et al., 2014). Then a rapid backward swimming of the predator was observed, leading to a separation of the ciliates, likely concomitant with protrichocysts ejection (Supplementary Fig. S1A–D). If the attacked *P. cristata* cells were not picked up immediately, they gradually slow down moving and were torn apart and partially eaten by the predators within five minutes (Supplementary Fig. S1E–H). By SEM, the ejection of protrichocysts was observed in both the anterior (apparently not a massive ejection) and the posterior (a massive ejection) portions of *P. cristata* cells in three replicates (Fig. 2D–G), with protrichocysts ejection rate of 80 %, 80 % and 90 %, respectively (Fig. 2H). On the contrary, in the control group (i.e., cells not exposed to predators), no protrichocysts ejection was observed under the same experimental conditions (Fig. 2C, H).

### Ejecting process of protrichocysts and histochemical analyses

**Fig. 3:**
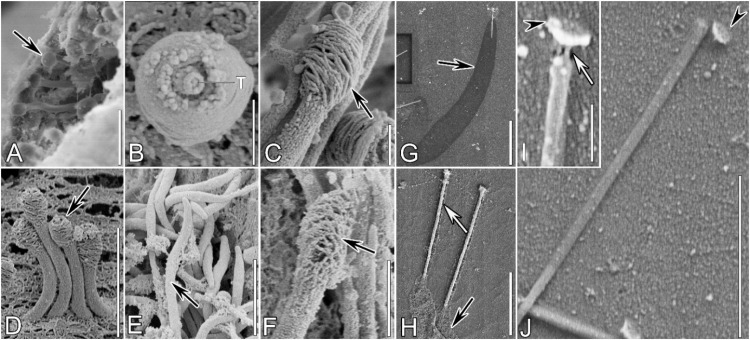
Scanning electron micrographs of protrichocysts of *Pseudourostyla cristata* in the resting state (**A**) and during ejection (**B‒I**) after induction by a methyl green pyronin treatment (see main text for details). **(A)** Resting state protrichocysts beneath the pellicle when cell was pricked after SEM preparation; a clear cap structure can be observed (black arrow). **(B)** An extruded protrichocyst with the tip (T) exposed out of the pellicle, surrounded by the cap**. (C, D)** Protrichocysts with the body partially ejected, showing a loose microtubule-like structure of the cap (black arrows). **(E, F)** The less dense posterior part of the body exploding on the cell surface (black arrow) and the microtubule-like circles of the cap gradually decomposing, before protrichocysts leave the cell (black arrow). **(G, H)** After the explosion of the less dense posterior part of the body (black arrow), the shaft is pushed out from it and wrapped around by the dense anterior part of the body (white arrow). **(I)** The dense anterior part of the body disintegrates, and the tip (arrowhead) is about to disconnect: a filamentous connecting structure is visible (white arrow); **(J)** The tip (arrowhead) separates from the shaft. Scale Bars = 5 μm (**F**); 3 μm (**A, D, E, G**); 1 μm (**H, I**); 0.5 μm (**B, C**); 0.1 μm (**J**).

**Fig. 4:**
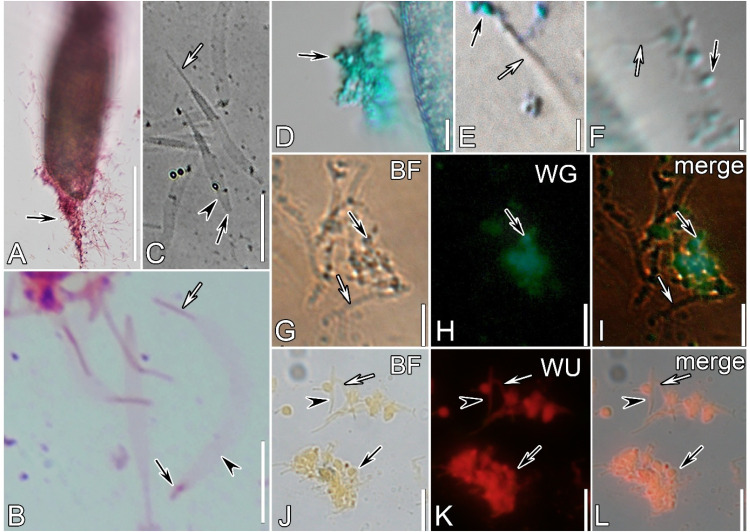
Bright field **(A-G, J)** and fluorescence microscope **(H, I, K, L)** micrographs of protrichocysts extrusion and components in *Pseudourostyla cristata* after treatment with different chemicals. Ejection induction by methyl green-pyronin (**A, B, G-I**), calcium chloride-alcian blue (**C, D-F)**, and acridine orange (AO) (**J-L**) respectively (see main text for details on the treatments). (**A**) methyl green pyronin induced a more violent protrichocysts ejection, namely with the maximal ejection distance and a full morphological extension of the extrusome; black arrow indicates violently ejected protrichocysts. **(B, C)** Ejected protrichocysts are away from the cell with an exploded body (arrowheads); white arrows indicate the central shaft maintaining the same length as in resting state, while black arrows indicate the spherical anterior ends depolymerized. **(D‒F)** Protrichocysts’ ejection stimulated by calcium chloride-alcian blue, to show that the spherical anterior end (black arrows) was stained while the body (white arrows) was not. **(G‒I)** After methyl green-pyronin treatment, FLUTAX-II labeling showing the presence of an acidic component and microtubules in the anterior parts of protrichocysts (black arrows) ejected, but not in the body part (white arrows). (**J**) Aggregated caps of protrichocysts after AO-induced ejection are indicated by black arrows. **(K, L)** AO treatment labeled the entire protrichocyst, including the cap (black arrows), the body (white arrows), and the central shaft (arrowheads), indicating the presence of an acidic and/or a phosphorylated protein component. Channels: BF, bright field; WG, ex/em: 492/520 nm; WU, ex/em: 460/650 nm. Scale Bars = 50 μm (**A**); 10 μm (**B‒D, G‒L**); 5 μm (**E, F**).

Methyl green–pyronin elicited maximal ejection, driving protrichocysts to their greatest distance from the cell (up to ∼200 µm) and effecting complete morphological extension (Figs. 3, 4A–B), thus this treatment was used for further observation of morphological details during the ejecting process. The resting protrichocysts beneath the pellicle (Fig. 1A) showed a rod-like shape, a size of 3 μm × 0.8 μm in size (at the widest anterior part) and a cap at its anterior end (Fig. 3A).

At an early stage of ejection, only the cap or part of the body extruded from the pellicle, and an orderly coiled microtubule-like structure with a diameter of about 20 nm in the cap and the tip at the center were exposed (Fig. 3B). Next, the body part of protrichocyst was obviously elongated; while the microtubule-like structure in the cap became loosely arranged, and 20–28 coils, about 25 nm in diameter each, were visible (Fig. 3C, D). The morphology and location of the tip did not change compared with the resting state (Figs. 1A, 3A). After the less dense posterior part of the body exploded on the cell surface (Fig. 3E) the microtubule-like structure became disordered as the cap had disintegrated (Fig. 3F).

Meanwhile, the body gradually extended up to 20–30 μm in length (i.e., it became 7–10 times as long as the resting state) and 3 μm in width (Figs. 3G, 4C); the shaft did not change in size compared to its resting state, but it seemed to be gradually pushed out of the exploded less dense body part (Figs. 3G, H; 4C). Observing the ejected protrichocysts, it was found that the shaft was enveloped by a thin layer of transverse fibrous material, which may correspond to the dB. There are horizontal fibers and fine meshlike substances at the back end of the protrichocysts, which might be the lB (Fig. 3H). Supposedly, it is the dense body that pushes the central shaft out, and the nail-head shaped tip might assist it in driving the shaft ejection. With the disintegration of the dB, the central shaft was completely exposed, and the tip was separated from the central shaft at this time (Fig. 3I). A filamentous connection between the tip and the top of the shaft could sometimes be observed (Fig. 3J).

The ejection of protrichocysts completed in a short time and achieved a distance of hundreds of microns after cells were stimulated by all the three chemical treatments used, i.e., methyl green-pyronin, calcium chloride-alcian blue, and acridine orange (AO) treatments (Fig. 4A–C, J). The protrichocysts’ morphological changing process during their ejection, i.e., the body extension followed by the shaft explosion (Fig. 4B, C, J), did not diverge among the three different treatments. However, protrichocysts extrusion induced by using methyl green-pyronin and calcium chloride-alcian blue solutions led to an extension up to 20 μm (*n*= 10) and no distinct spherical structures were observed at the anterior end of the protrichocysts (Fig. 4B, C), while the one induced by AO was below 10 μm (*n* = 10) and some distinct spherical structures were observed at the organelle’s anterior end (Fig. 4J).

In the extruded protrichocysts induced by calcium chloride-alcian blue treatment, the spherical anterior end was stained while the body was not (Fig. 4D–F), indicating the local presence of an acidic component highlighted by the alcian blue. The presence of microtubules was detected by means of FLUTAX-II fluorescence only on the spherical anterior of protrichocysts extruded after the methyl green-pyronin treatment (Fig. 4G–I); no fluorescence resulted in the control group (not shown). Finally, the whole protrichocysts were stained by the treatment with AO, as indicated by a yellow color under the bright field microscope and a distinct orange-red color under the fluorescence microscope (Fig. 4J–L); these results indicate a diffused presence of an acidic and/or a phosphorylated protein component.

### Molecular analysis of the main proteins of protrichocysts

#### Protein bands

**Fig. 5:**
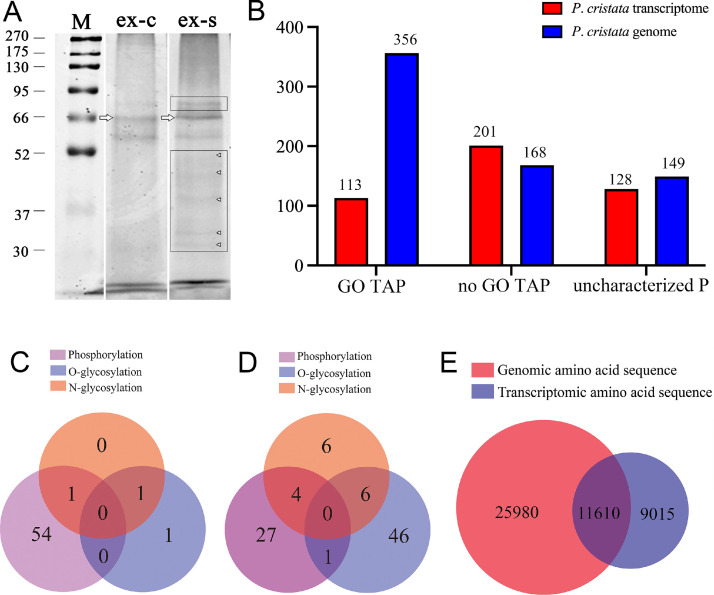
Molecular analysis of the main proteins of the protrichocysts. **(A)** SDS-PAGE analysis of the proteins; ex-c, Coomassie brilliant blue staining, ex-s, silver re-staining of the gel stained by Coomassie brilliant blue after decolorization, M, marker; the black rectangles show the additional protein bands (arrowheads) revealed by silver staining (a band between 66 and 95 kDa and five faint bands between 30 and 52 kDa), while arrows indicate the band (about 66 kDa) used for HPLC-MS/MS analysis. **(B)** Overview of protrichocysts main proteins obtained by means of HPLC-MS/MS identified by searching against the *P. cristata* transcriptome database and genome database. The uncharacterized proteins contain unknown proteins, hypothetical proteins, putative proteins, and predicted proteins. Y-axis: protein number. GO, Gene Ontology. GO TAP, GO terms annotated proteins. no GO TAP, no GO terms assigned proteins. uncharacterized P, uncharacterized proteins. **(C, D)** Modification of protrichocysts main proteins identified by searching against the *P. cristata* transcriptome database (**C**) and genome database **(D),** respectively. **(E)** Cluster analysis of amino acid sequence from the *P. cristata* transcriptome database and genome databases.

**Fig. 6:**
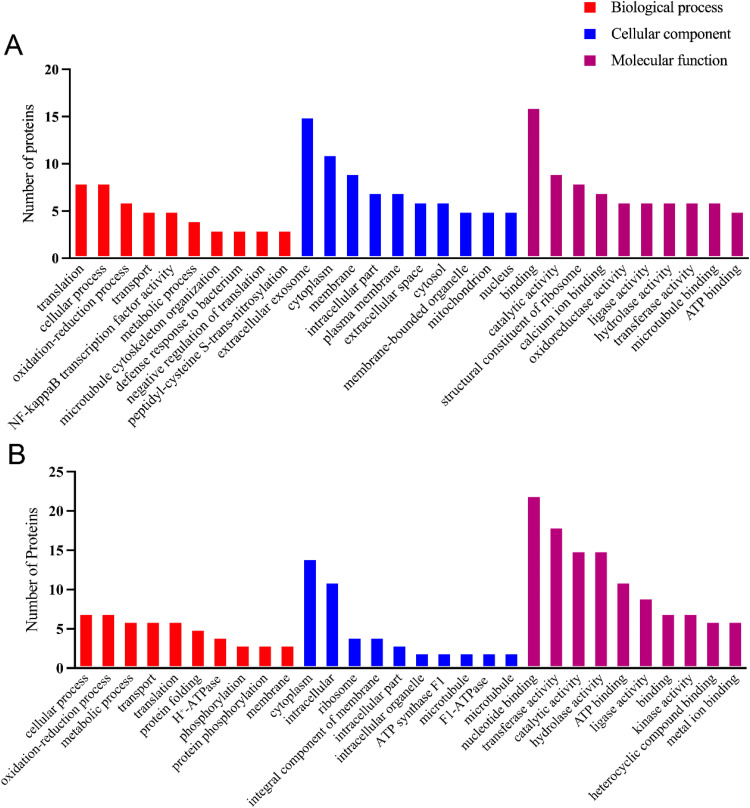
The top ten terms in each Gene Ontology category, ranked by the number of annotated protrichocysts proteins identified by searching against the *P. cristata* transcriptome database **(A)** and genome database **(B)**. H⁺-ATPase, ATP hydrolysis coupled proton transport; ATP synthase F1, proton-transporting ATP synthase complex catalytic core F (1); F1-ATPase, proton-transporting two-sector ATPase complex catalytic domain.

HPLC-MS/MS offers high sensitivity. However, reliable protein identification requires that the target proteins are present in sufficient abundance, well separated, and supported by existing functional databases (Neubert et al., 2020). Consequently, as the whole proteome analysis of protrichocysts failed to identify proteins enriched in exocytosis-related terms, we initiated a study starting with protein separation by gel electrophoresis. For the molecular analysis of protrichocyst proteins (Table 1; Figs. 5, 6), we first analyzed the Coomassie brilliant blue-stained gel (Fig. 5A), where two distinct bands were observed between molecular weights of 52 kDa and 66 kDa. Upon re-staining with silver, a new, prominent band was detected between 66 kDa and 95 kDa, and five faint bands appeared between 30 kDa and 52 kDa (Fig. 5A). We focused on the 66 kDa band, as it was the most prominent and distinct band in the Coomassie brilliant blue-stained gel, making it the best candidate for further analysis.

#### Protrichocysts proteins characterization

We identified 442 credible proteins by searching against the *P. cristata* transcriptome database, including 314 functional proteins (113 GO terms annotated proteins, 201 unannotated [no GO terms assigned] proteins) and 128 uncharacterized proteins (Fig. 5B). We identified 673 credible proteins by searching against the *P. cristata* genome database, including 524 functional proteins (356 GO terms annotated proteins, 168 unannotated proteins) and 149 uncharacterized proteins (Fig. 5B).

#### Functional categories by gene ontology

In each category, the ten most abundant terms were ranked according to the number of annotated proteins for proteins identified from the two databases (Fig. 6A–B). When searching against the *P. cristata* transcriptome, the top three terms mapped to translation, cellular process and oxidation–reduction process in BP category, extracellular exosome, cytoplasm and membrane in the CC category, binding, catalytic activity and structural constituent of ribosome in the MF category (Fig. 6A). When searching against the *P. cristata* genome, the top three terms mapped to cellular process, oxidation–reduction process and metabolic process in BP category, membrane, cytoplasm and intracellular in CC category, nucleotide binding, transferase activity and catalytic activity in MF category (Fig. 6B).

#### Phosphorylation and glycosylation modification

*P. cristata* showed a large gene family expansion enriched for protein phosphorylation and phosphorelay signal transduction systems (Jin et al., 2024). Our fluorescence staining results with AO indicate the possible presence of phosphorylated protein components. Given that post-translational modifications such as phosphorylation and glycosylation profoundly influence protein function, localization, and interactions (Duan and Walther, 2015; West and Kim, 2019), their identification was therefore undertaken in this study. Phosphorylation and glycosylation (including N-glycosylation and O-glycosylation) modifications were detected by searching against the *P. cristata* transcriptome (Fig. 5C) and genome databases (Fig. 5D)*.* Against the *P. cristata* transcriptome, 55 proteins carried phosphorylation, two bore O-glycosylation, and two bore N-glycosylation (Fig. 5C); against the *P. cristata* genome, the counts rose to 32, 53, and 16, respectively (Fig. 5D). Moreover, 69 % of the *P. cristata* transcriptome-derived proteins were acidic (pI < 7) versus 31 % basic (pI > 7).

A cluster analysis revealed that the transcriptomic amino acid sequences had 9015 different clusters than the genomic amino acid sequences (Fig. 5E). This disparity likely explains the higher recovery of phosphoproteins and the exclusive detection of extracellular-exosome proteins in the *P. cristata* transcriptome compared with its genome.

#### Proteins related to extracellular exosome

By searching against the *P. cristata* transcriptome database we identified 15 proteins annotated to extracellular exosome (GO:0070062) in CC category (Table 1). Most of them were predicted or putative proteins, and six possessed a peptide number of two or more: alpha tubulin (putative), S100 calcium binding protein A9 (partial), actin, cytoplasmic 2 (predicted), glyceraldehyde-3-phosphate dehydrogenase isoform 2, guanine nucleotide-binding protein subunit beta-2-like 1, and lysozyme C precursor. The unique peptides and sequence coverage of the putative alpha tubulin were the highest.

**Table 1:** Protrichocyst proteins annotated to the term extracellular exosome [GO:0070062] searching against transcriptomic database of *Pseudourostyla cristata*.

Protein ID	Annotation		Unique peptides	Sequence coverage [%]	Molecular weight [kDa]
TRINITY_DN20641_c0_g1_i1		Alpha tubulin, putative	18	50	49.619
TRINITY_DN25783_c0_g1_i1		S100 calcium binding protein A9, partial	5	39.5	13.242
TRINITY_DN19180_c0_g1_i1		PREDICTED: actin, cytoplasmic 2	4	14.4	38.244
TRINITY_DN10310_c0_g1_i1		glyceraldehyde-3-phosphate dehydrogenase isoform 2	2	13.4	23.119
TRINITY_DN16997_c0_g1_i2		guanine nucleotide-binding protein subunit beta-2-like 1	2	6.7	37.274
TRINITY_DN19474_c2_g1_i2		lysozyme C precursor	2	14.2	16.537
TRINITY_DN11322_c0_g1_i1		PREDICTED: uncharacterized protein LOC101381131	1	8.8	12.567
TRINITY_DN11988_c0_g1_i1		glyceraldehyde-3-phosphate dehydrogenase	1	6.3	35.704
TRINITY_DN14000_c0_g3_i1		PREDICTED: 40S ribosomal protein S3 isoform X1	1	10.3	13.607
TRINITY_DN15352_c0_g1_i1		E2 ubiquitin-conjugating enzyme variant	1	10.1	15.651
TRINITY_DN17947_c0_g1_i1		ubiquitin ribosomal protein s27ae fusion protein	1	34.1	14.21
TRINITY_DN27377_c0_g1_i1		PREDICTED: serine/threonine-protein phosphatase 2A 65 kDa regulatory subunit A alpha isoform	1	2.5	64.529
TRINITY_DN34896_c0_g1_i1		L-plastin variant, partial	1	5.5	26.147
TRINITY_DN37390_c0_g1_i1		PREDICTED: ubiquitin-40S ribosomal protein S27a	1	26.4	18.553
TRINITY_DN38386_c0_g1_i1		PREDICTED: protein S100-A8	1	10.5	12.253

Additionally, searches against the two databases also recovered a variety of heat shock proteins (HSPs) (Table 2) which are established exosome markers in metazoans (Griffiths et al., 2019).

**Table 2:** Heat shock proteins (established exosome markers from literature) identified by searching against the transcriptomic and genomic databases of *Pseudourostyla cristata*.

Database	Protein ID	Annotation	Unique peptides	Sequence coverage [%]	Molecular weight [kDa]
*P. cristata* transcriptome	TRINITY_DN28094_c0_g1_	Heat shock protein 70	17	35.6	67.906
	TRINITY_DN19291_c0_g1_i1	Heat shock protein 90	17	25.2	82.258
*P. cristata* genome	g16920.t1	Heat shock protein 70	22	35.6	71.894
	g21778.t1	Heat shock protein 90	19	32.6	71.07
	g8585.t1	Heat shock protein 90	17	25.2	82.258
	g25994.t1	Heat shock protein 90	5	10.6	99.438
	g12579.t1	Heat shock protein 70	2	2.3	94.604
	g6271.t1	Heat shock protein 70	1	1.2	106.37

### The regeneration cycle of protrichocysts and the associated genes

Following stimulation with AO, nearly all protrichocysts in *P. cristata* were ejected (Supplementary Fig. S2A, B). Our time-course analysis revealed that protrichocysts density progressively increased and fully recovered to vegetative stage levels by 12 h (Supplementary Fig. S2C–F). Based on this regeneration dynamics profile, we selected the 6 h post-treatment time point, representing the regeneration state group (RS), for a subsequent transcriptional analysis of regeneration-associated gene expression.

**Fig. 7:**
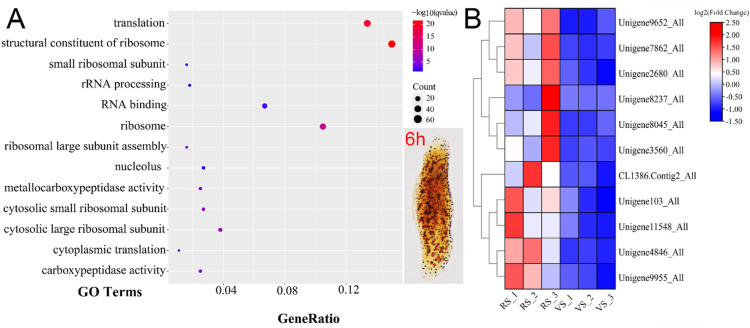
Functional characterization of upregulated genes during *P. cristata* protrichocysts regeneration (6 h post-treatment). **(A)** Significantly enriched Gene Ontology (GO) terms (*p*-value < 0.1) of the regeneration-upregulated unigenes. Inset: protargol-stained cell at 6 h post-treatment—the regeneration stage (RS) selected for transcriptional analysis. **(B)** Expression patterns of 11 carboxypeptidase-associated genes. RS_1–3, regeneration stage replicates 1–3; VS_1–3, vegetative stage replicates 1–3.

The replicates of cells in regeneration and vegetative phases clustered into two separated groups in PCA analysis (Supplementary Fig. S3A). We identified 3947 differentially expressed genes (DEGs), including 1441 upregulated and 2506 downregulated genes (Supplementary Fig. S3B). GO term enrichment analysis identified 294 upregulated unigenes and categorized them into one or more GO categories, i.e., Biological Process (BP), Cellular Component (CC), Molecular Function (MF) (Supplementary Table S1, Fig. 7A). The most abundant MF annotations for these DEGs were structural constituent of ribosome, RNA binding, metallocarboxypeptidase activity, carboxypeptidase activity; annotations in CC were ribosome, cytosolic large ribosomal subunit, cytosolic small ribosomal subunit, and nucleolus; and annotations in BP were translation, rRNA processing, ribosomal large subunit assembly, cytoplasmic translation (Fig. 7A, Supplementary Fig. S4C). Additionally, 64 upregulated DEGs in 6 h post-treatment cells were enriched in genetic information processing in Kyoto Encyclopedia of Genes and Genomes (KEGG) database (Supplementary Fig. S4D). Notably, 11 carboxypeptidase-related genes (including Unigene8237_All, Unigene8045_All, Unigene103_All, CL1386.Contig2_All, Unigene11548_All, Unigene7862_All, Unigene3560_All, Unigene4846_All, Unigene9652_All, Unigene9955_All, Unigene2680_All) were co-upregulated alongside ribosomal markers (Fig. 7B).

## Discussion

### Dual-phase protrichocyst ejection: a novel extruding mechanism bridging trichocysts and toxicysts

**Fig. 8:**
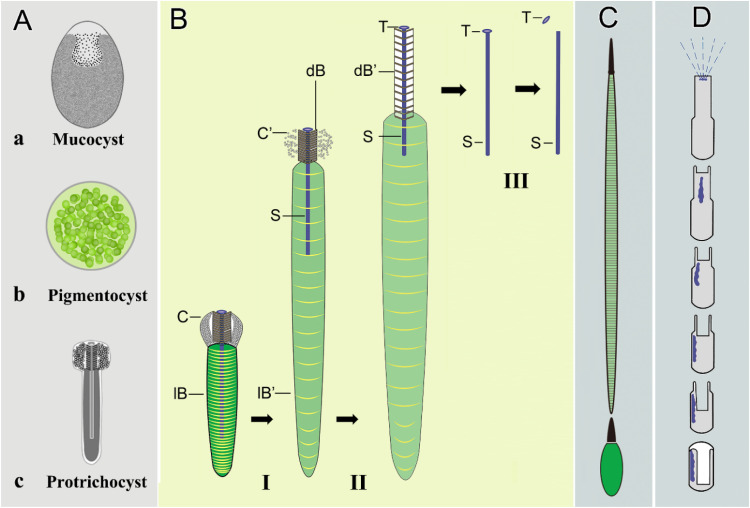
Schematic diagrams of the protrichocysts and few other very diffused and well-known extrusomes for a comparison of organelle structure and ejection process. (**A**) Three major extrusome types among the subclass Hypotrichia: protrichocyst exhibits the most complicated structural organization compared with mucocyst and pigmentocyst. **(B)** The ejection process of protrichocysts reconstructed in three-stages: (Ⅰ) the less dense posterior part of the body (lB) extends (lB’) and the cap (C) dissolves (C’); (II) the dense anterior part of body (dB) extends (dB’) and the shaft (S) is pushed out with the help of the tip (T), which may supply the force application point; (ⅡI) the less dense posterior part of the body disintegrates, the dense anterior part of body dissolves and the tip (T) separates from the shaft (S). **(C, D)** The ejection of the two most known projectile extrusomes in ciliates, i.e., the trichocysts (**C**) and the toxicysts (**D**): the body extends during the ejection process of trichocysts (Hausmann, 1978), while there is a protrusion of the casing structure and a toxin release in toxicysts (Wolf, 2014).

Within the subclass Hypotrichia, mucocysts and pigmentocysts (Fig. 8A) are ubiquitous extrusomes that undergo slow, passive release with negligible structural reconfiguration. In contrast, protrichocysts display explosive ejection dynamics, involving three violent restructuring stages: (I) the microtubule-like structure of the cap gradually loosens and disintegrates, with the less dense posterior part of the body (lB) extending dramatically; (II) the dense anterior part of the body (dB) extends, pushing the central shaft out of body; (III) the tip separates from the shaft and the central shaft is exposed (Fig. 8B). Among ciliate extrusomes, spindle-shaped trichocysts and toxicysts are renowned for their dramatic morphological transformations during ejection (Bannister, 1972; Wolf, 2014), serving as key comparators for the explosive ejection dynamics of protrichocysts (Fig. 8C, D).

Trichocysts mainly exist in species belonging to the order Peniculida (e.g., *Paramecium, Frontonia*). They are distinctively composed of a body and a tip. In *Paramecium*, the body experiences an eight-time stretching during the discharging process while the tip remains in its original shape and does not break away from the body (Rosati and Modeo, 2003); local trichocyst exocytosis provides a chance for *Paramecium* to escape by means of a rapid propulsion away from the attacking predator. The discharging process of protrichocysts is similar to that of trichocysts in terms of the extension and stretching of the body part, whereas the body extension of the former likely happens twice (due to the extension of lB and dB) aiming at pushing the central shaft out, which does not occur in the latter. Additionally, the nail-head shaped tip may supply the force application point for dB extension pushing the shaft out, and it separates from the shaft when the ejection process finishes. Although there is a sheath composed of a microtubule-like structure covering the tip of trichocysts as well, it never expands, or dissolves as seen in protrichocysts. Therefore, from the perspective view of the ejection process, we can confirm that protrichocysts are a distinct extrusive organelle compared to trichocysts (Suganuma, 1973; Zhang et al., 2011).

Predator ciliates of the subclass Haptoria and the class Prostomatea possess toxicysts. Although several subtypes have been discovered, they are in general used to help predation by releasing toxic substances (Hausmann, 1978). Toxicysts show a telescopic tube structure with toxins included. When ejected, the telescopic tube is inverted, and toxin is released at the same time (Wolf, 2014). Though their morphology in the resting state is not easily confused, our results indicated the final pushing-out of the shaft in protrichocysts is somewhat similar to the excretion of toxic substance from the toxicysts, i.e., both the extrusomes expose the substances that are tightly wrapped inside in their resting state. In the case of protrichocysts, the cap may function to prevent the extension of the dB and the exposure of the shaft before the time is ripe.

Previous studies speculated that the ejection of protrichocysts may be a stress reaction providing self-protection based on a fixative-stimulated ejection (Grim and Manganaro, 1985; Zhang et al., 2011). In the present study, we revealed that the discharging process of protrichocysts resembles that of trichocysts (though with differences in details) in ejecting a bar-shaped structure, which might support the idea that the defense function of protrichocysts is achieved by creating a physical space as in the case of trichocysts (Bilinski et al., 1981; Knoll et al., 1991). Furthermore, protrichocysts and toxicysts have a common final result of ejection, i.e., the exposure of a substance contained in the resting state organelle for attacking as discussed above (Wolf, 2014). We also found some proteins related to chemical defense from the protein identification of protrichocysts’ ejected structure (see the following discussion). Therefore, we speculate that protrichocysts exhibit a unique hybrid defense mechanism, integrating the mechanical projection of trichocysts with the chemical secretion characteristic of toxicysts. The backward swimming of the predator after attacking the prey during the predator-prey interaction, seems to provide additional support for the defense function of protrichocysts. Therefore, future studies that can confirm the localization of these toxic proteins within the central shaft and establish their similarities to toxicysts would be essential to ultimately validate our hypothesis regarding this integrated chemical defense mechanism.

### Potential chemical defense function of protrichocysts inferred from the proteins associated with extrusomes

Based on functional studies on ciliates’ extrusomes, secondary metabolites contained in the pigmentocysts were proved to provide a chemical defense in several ciliate groups (Alimenti et al., 2022). Examples include climacostol, blepharismins, and mono-prenyl hydroquinone in the pigmentocysts of heterotrichs (Buonanno et al., 2012; Harumoto et al., 1998; Miyake et al., 2003), as well as keronopsins, and erythrolactones of kentrurostylids (Table 3) (Anesi et al., 2016; Höfle et al., 1994). As for the keronopsamides (Table 3) (Guella et al., 2010), the chemical defense role is still to be fully demonstrated, although it is considered highly probable (Modeo L., *pers. commun*).

**Table 3:** Comparison of protrichocysts, pigmentocysts, and mucocysts found in various ciliates of the subclass Hypotrichia focusing on their components and their proposed functions.

Species	Types	Components	Functions	Data source
*Pseudourostyla cristata*	Protrichocysts	Glycosylated/phosphorylated proteins	Mechanical/ chemical defensec ell-cell communication environmental sensing and adaptation	(Suganuma, 1973); Present study
*Pseudourostyla nova*	Protrichocysts	‒		(Zhou et al., 2011)
*Anteholosticha monilata*	Protrichocysts	‒		(Zhang et al., 2012)
*Pseudokeronopsis erythrina*	Pigmentocysts	Erythrolactones	Chemical defense	(Anesi et al., 2016)
*Pseudokeronopsis riccii*	Pigmentocysts	Keronopsamides*		(Guella et al., 2010)
*Pseudokeronopsis rubra*	Pigmentocysts	Keronopsins		(Höfle et al., 1994)
*Pseudokeronopsis carnea*	Pigmentocysts	‒		(Wirnsberger and Hausmann, 1988)
*Thigmokeronopsis jahodai*	Pigmentocysts	‒		(Wicklow, 1981)
*Diaxonella pseudorubra*	Pigmentocysts	‒		(Sun et al., 2014)
*Paraurostyla weissei*	Pigmentocysts	‒		(Jerka-Dziadosz, 1982)
*Engelmanniella mobilis*	Mucocysts	‒	Cell-cell communicatione nvironmental sensing and adaptation	(Wirnsberger-Aescht et al., 1989)
*Urostyla grandis*	Mucocysts	‒		(Bardele, 1981) (Zhang et al., 2007)
*Architricha indica*	Mucocysts	‒		(Zhang et al., 2014)
*Neogastrostyla chongmingensis*	Mucocysts	Glycoproteins		(Lu et al., 2025)
*Oxytricha granulifera*	Mucocysts	Glycoproteins		(Lu et al., 2025)
*Pseudoamphisiella lacazei*	Mucocysts	‒		(Cai et al., 2017)
*Urosoma emarginata*	Mucocysts	‒		(Dong et al., 2020)
*Urosoma salmastra*	Mucocysts	‒		(Dong et al., 2020)

“‒”, data not available; * the chemical defense is still to be fully demonstrated, although considered highly probable (see main text).

In the case of protrichocysts the analysis of their credible proteins annotated to the entry of extracellular exosomes (Table 1) may further support their hypothetical role in the cell chemical defense on the basis of several details of the ejection process. First, proteins that may be related to defense against bacteria were identified, namely S100 A8 and S100 A9. They belong to the S100 family proteins and have high affinity for divalent ions (such as Ca^2+^and Zn^2+^), usually existing in the form of heterodimers (Harumoto et al., 1998). The S100A8/A9 complex is involved in inflammation, cell proliferation, differentiation, apoptosis, and exhibits a broad-spectrum antimicrobial activity against numerous microorganisms, such as *Escherichia coli, Candida albicans* and *Listeria monocytogenes* (Petrelli et al., 2012; Xia et al., 2024). Second, glyceraldehyde-3-phosphate dehydrogenase (GAPDH) plays various physiological roles in infection via secretion in both protozoa and bacteria. In the parasitic protozoan *Leishmania major*, GAPDH is secreted by extracellular vesicles and can suppress the expression of host protein TNF-α which can activate infected macrophages to destroy *Leishmania* or interact with other cells and lymphokines of the immune system resulting in an enhanced resistance to it (Aggarwal and Natarajan, 1996; Das et al., 2021; Ejghal et al., 2021; Schiopu and Cotoi, 2013). In bacteria, GAPDH secreted by extracellular vesicles could affect pathogenesis and might facilitate the long-term contact between bacterial and epithelial cells, causing increased epithelial cell/tissue damage (Clark et al., 2016).

### Protrichocysts composition reveals its evolutionary divergence and convergence with other protist extrusomes

Histochemical methods are successfully applicable to study a variety of biological problems and can give simultaneously biochemical and morphological information. However, reproducible positive results are considered to be reliable and significant to shed light on chemical composition and activities of tissues, while negative results cannot be taken as definitive (Rosati and Gabrielli, 2005). In this study, by the experimental induction of protrichocysts’ ejection, we still gained information to start understand the fundamental components of protrichocysts.

The positive staining of protrichocysts cap with alcian blue indicated that the cap contains acid mucopolysaccharides (Møller and Poulsen, 2009). Meanwhile, the staining with acridine orange (AO) indicated that either phosphorylated protein or acid mucopolysaccharides might be distributed over the whole protrichocysts given that they present an overall orange-red fluorescence (Anderson and Orci, 1988; Klut et al., 1985; McMaster and Carmichael, 1977; Mellman et al., 1986). Considering the discrepancy of the results from the two staining methods, there are two possibilities in terms of the general components of protrichocysts: (1) protrichocysts contain phosphorylated proteins with acid mucopolysaccharides concentrated in the cap. Both possibilities suggest a more complex histochemical composition of the cap compared to the other parts of the protrichocysts. This is also supported by the results of Flutax-II labelling, which detected the alpha tubulin in the separated protrichocysts protein confirming that microtubules are present in the cap (Suganuma, 1973); (2) the whole protrichocysts contain acid mucopolysaccharides with extra kinds of them distributed only in the cap;

A positive staining with alcian blue has also been observed in other extrusomes of diverse protists. For examples, the mucocysts of *Tetrahymena* and the horn-shaped extrusomes of *Oxytricha* (among ciliates) (Glas-Albrecht and Plattner, 1990; Tang et al., 2016; Wolfe, 1988), the trichocysts and the nematocysts of the dinoflagellates *Oxyrrhis marina* and *Polykrikos kofoidii* respectively, the ribbon-like ejectisomes of the chlorophyte *Pyramimonas parkeae*, and the extrusomes of the actinophryd *Actinophrys sol* (among non-ciliate protists) (Leys and Hejnol, 2021; Rhiel et al., 2019; Yamagishi et al., 2015). This indicates a commonality in general composition of acid mucopolysaccharides among various extrusomes of distantly related protists. However, it is worth noting that, in most cases, there was an overall staining of the organelles, while in both the case of the protrichocysts of *P. cristata* and the nematocysts of *P. kofoidii* (a predatory dinoflagellate), only a portion of the extrusome was stained. The nematocysts of *P. kofoidii* consist of different portions, i.e., a capsule, a coiled tubule, a stylet, and an operculum. Only the tubule, which was suggested to act as a soluble delivery system when injected into the prey during predation by the dinoflagellate, and the everted portion of the capsule were stained by alcian blue (Leys and Hejnol, 2021). This result suggests the cap of protrichocysts might play a role in cell-to-cell or cell-to-organism interaction as well, with the help of the substances targeted by the alcian blue.

Microtubules are mainly involved in cytoskeleton formation in eukaryotic cells (Duan and Walther, 2015). Among the extrusomes studied in ciliates, microtubule-like structures were found in the sheath covering the tip of the trichocysts of *Paramecium* (Wichterman, 1986), and in the sheets separating rows of trichites of *Strombidium* (Modeo et al., 2001). The presence of microtubules in protrichocysts’ structure renders it a unique case among protists’ extrusomes, but the role of microtubules in protrichocysts’ function has been incomprehensible so far (Bardele, 1969; Hausmann, 1978).

In the special proteome analysis, the abundance of acidic proteins and proteins that had undergone glycosylation and phosphorylation further supported the presence of both the histochemical results. As the major types of post-translational modifications of proteins, phosphorylation and glycosylation confer proteins more diverse cellular functions in eukaryotic cells (Duan and Walther, 2015; West and Kim, 2019). In extrusomes, the presence of glycoproteins and phosphoproteins is often reported (Folgueira et al., 2019; Glas-Albrecht et al., 1990; Idriss, 2001; Lu et al., 2025; Sakaguchi et al., 2001), while their function was only occasionally studied. A 40 kDa glycoprotein found in the extrusome of the actinophryd *Actinophrys sol* was confirmed to be evolved in recognizing prey and cell-to-cell interaction (Sakaguchi et al., 2001). It was therefore further supported that protrichocysts may have additional functions besides the cell defence.

In conclusion, protrichocysts share conserved acid mucopolysaccharides and glycoproteins with other extrusomes in general, yet uniquely localize microtubules and proteins to their caps, reflecting functional convergence and divergence during evolution.

### Evolutionary similarities: ciliates’ extrusomes and metazoans’ secretory granules

The formation of dense core granules (DCGs) involves multiple coordinated steps, including protein synthesis, post-translational modification, sorting, and condensation into mature secretory granules. Carboxypeptidases, a type of exopeptidase, also play a significant role in DCGs’ biogenesis. These enzymes catalyze the hydrolytic cleavage of amino acid residues from the carboxyl-terminal end of proteins/polypeptide chains (Vilijn et al., 1989), such as converting proinsulin to mature insulin and maturing neuropeptides (Solov'ev et al., 2016; Tokarz et al., 2018). In *Tetrahymena*, carboxypeptidases mediate post-translational trimming and cleavage of polypeptide chains, generating mature functional proteins of proGrl4p in mucocysts (Verbsky and Turkewitz, 1998). The marked upregulation of ribosome- and translation-related genes during protrichocyst formation in *P. cristata* indicates active protein synthesis, while the concurrent induction of carboxypeptidase-associated genes suggests that carboxypeptidases likely participate in the proteolytic processing of proteins within the protrichocysts’ dense core, thereby facilitating their physical and functional maturation.

It has been hypothesized that DCGs have emerged independently in metazoans and ciliates through convergent evolution (Thureson-Klein and Klein, 1990); however, the discovery that sortilins are required in the formation of secretory organelles via a receptor-mediated pathway in ciliates and apicomplexans, pointed instead to deep shared ancestry of secretory granules for diverse linages of eukaryotes (Briguglio et al., 2013). From the perspective of chemical composition of the inclusion, no similarities were discovered between protrichocysts and DCGs of metazoans, which is likely due to the functional specialization of the DCGs (e.g., DCGs secret hormones and neuropeptides in endocrine cells and neurons) (Kelly, 1985).

Unlike DCGs, which are regulated secretory granules derived from the Golgi apparatus, exosomes are endosome-derived extracellular vesicles (30–150 nm) that mediate intercellular communication. Similarities between protrichocysts and exosomes were also found. Firstly, proteins related to extracellular exosomes in protrichocyst were identified through a library search. Except for the proteins discussed above, HSP70 and HSP90 proteins are other most commonly used markers for exosomes of metazoans (Cho et al., 2005; Conde-Vancells et al., 2008; Lauwers et al., 2018). Secondly, AO selectively binds to single-stranded DNA (ssDNA) and RNA, yielding red fluorescence (McMaster and Carmichael, 1977). AO fluorescence (Fig. 4K) suggests that ssDNA or RNA might be present in the protrichocysts and a feature distinguishing exosomes from other biological granules is the presence of various types of nucleotides (e.g., microRNAs, mRNAs, long-stranded non-coding RNAs, small nuclear RNAs, piRNAs, tRNAs, and mitochondrial DNA) (Gajos-Michniewicz et al., 2014).

Overall, these findings suggest that evolutionarily conserved mechanisms are involved in the biogenesis and components of both ciliates and metazoans. Therefore, it is reasonable to speculate the similarities of fundamental functions in the different secretory pathways of protozoans and metazoan cells.

## Conclusions and future perspectives

This study, conducted on the hypotrich *Pseudourostyla cristata,* represents the first comprehensive investigation of protrichocysts, and offers three major breakthroughs: (1) by means of a predator-prey interaction experiment, the complex ejection mechanism was systematically characterized, stating the unique hybrid defense as the organelle’s primary function; (2) through a novel application of histochemical staining and HPLC-MS/MS, phosphorylated/glycosylated proteins and microtubular components within protrichocysts were identified, providing the first organelle’s molecular profile and suggesting additional roles potentially mediated through intercellular communication; and (3) by means of a comparative analysis, transcriptomic and proteomic similarities to metazoans were demonstrated, suggesting conserved secretory pathways across eukaryotes.

However, fundamental knowledge gaps remain to be filled: (1) the functional significance of protrichocysts’ structural components—the localization of secreted chemicals within protrichocysts remains unclear, particularly whether the shaft contains toxic proteins, which requires a direct validation; (2) the biogenesis pathway, including microtubule-dependent assembly mechanisms, which still needs to be characterized; and (3) beyond defense, the full ecological roles of protrichocysts and their functional validation in natural environments, which still needs to be understood. Addressing these challenges will require integrated approaches combining advanced imaging, functional genomics, and ecological experimentation. Such efforts will not only resolve long-standing questions about extrusome biology but also provide crucial insights into the evolution of eukaryotic secretory systems, positioning these organelles, which bridge mechanical projection and chemical secretion in ciliates, as a model for studying cellular innovation and adaptation.

## Funding

This work was supported by the National Natural Science Foundation of China (32170446, 32570525).

## Data availability

The datasets used and/or analyzed during the current study are available from the corresponding author on reasonable request.

## CRediT authorship contribution statement

**Kangqiao Dong:** Investigation, Writing – original draft, Writing – review & editing. **Peilin Cai:** Investigation, Validation, Writing – original draft, Methodology. **Liping Lyu:** Methodology, Formal analysis. **Juan Yang:** Methodology, Software, Formal analysis, Visualization. **Yi Wu:** Investigation, Writing – original draft. **Letizia Modeo:** Writing – review & editing. **Xiao Chen:** Methodology. **Jing Xu:** Writing – review & editing. **Xinpeng Fan:** Funding acquisition, Supervision, Project administration, Writing – review & editing, Resources, Conceptualization.

## Declaration of competing interest

**Research Support**: This research received no external financial or non-financial support.

**Relationships:** There are no additional relationships to disclose.

**Patents and Intellectual Property:** There are no patents to disclose.

**Other Activities:** There are no additional activities to disclose.

## Appendix Supplementary materials

Image, application 1
