# Supplementary material for: Protrichocysts: a hybrid defense extrusive organelle bridging mechanical projection and chemical secretion in ciliates
Source: Curr Res Microb Sci. 2025 Dec 23;10:100539. doi: 10.1016/j.crmicr.2025.100539 (PMC12811490; doi:10.1016/j.crmicr.2025.100539)
Supplement: Supplementary file 1 [file mmc1.docx]

**Supplementary material**

**
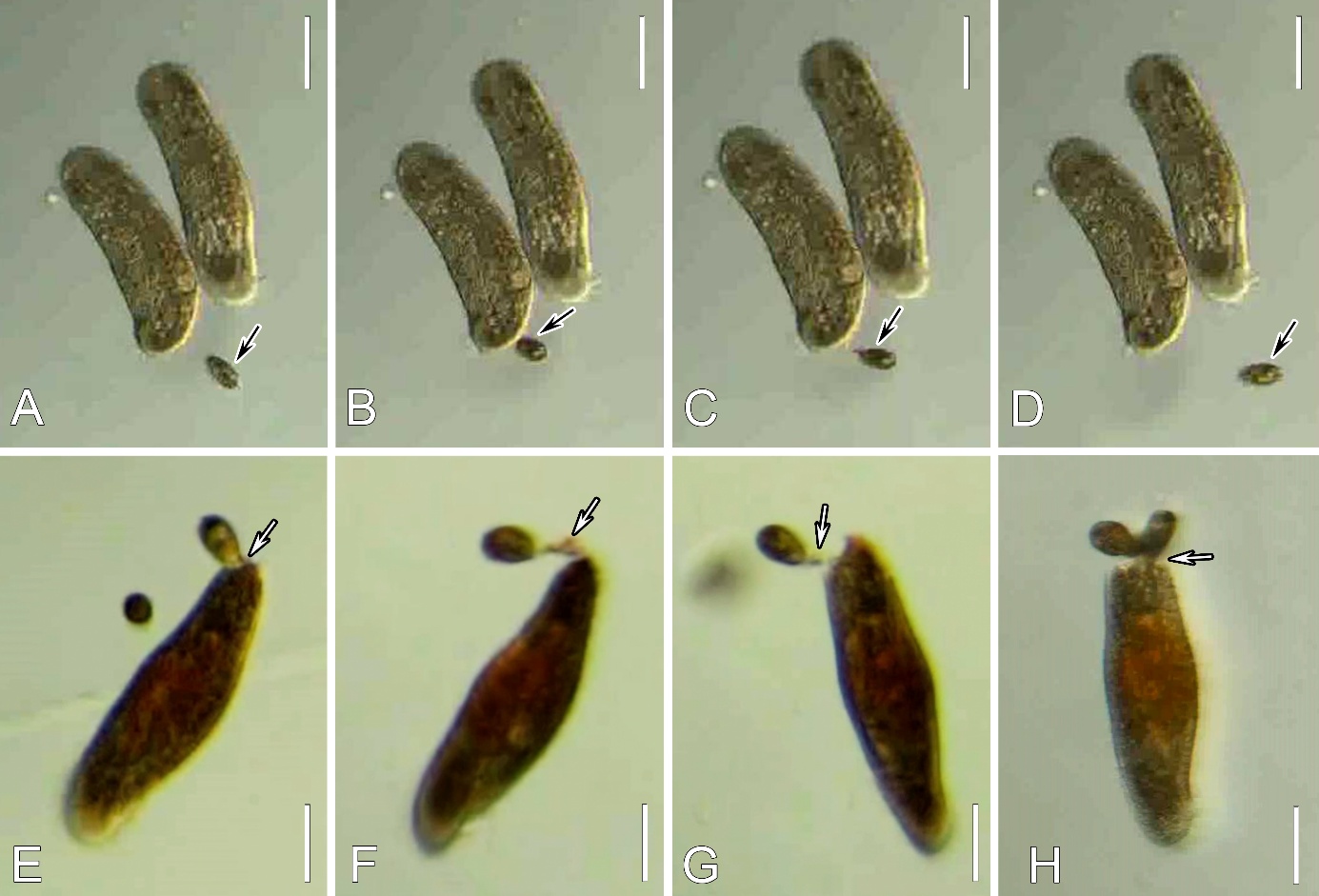
**

**Fig. S1** A rapid backward swimming of the predator was observed (black arrows), leading to a separation of the ciliates when *Coleps* attacked *P. cristata* with its anterior part **(A–D)**. *P. cristata* cells were partially eaten by predators (white arrows) within five minutes **(E–H)**. Scale bars = 50 μm (A–H)


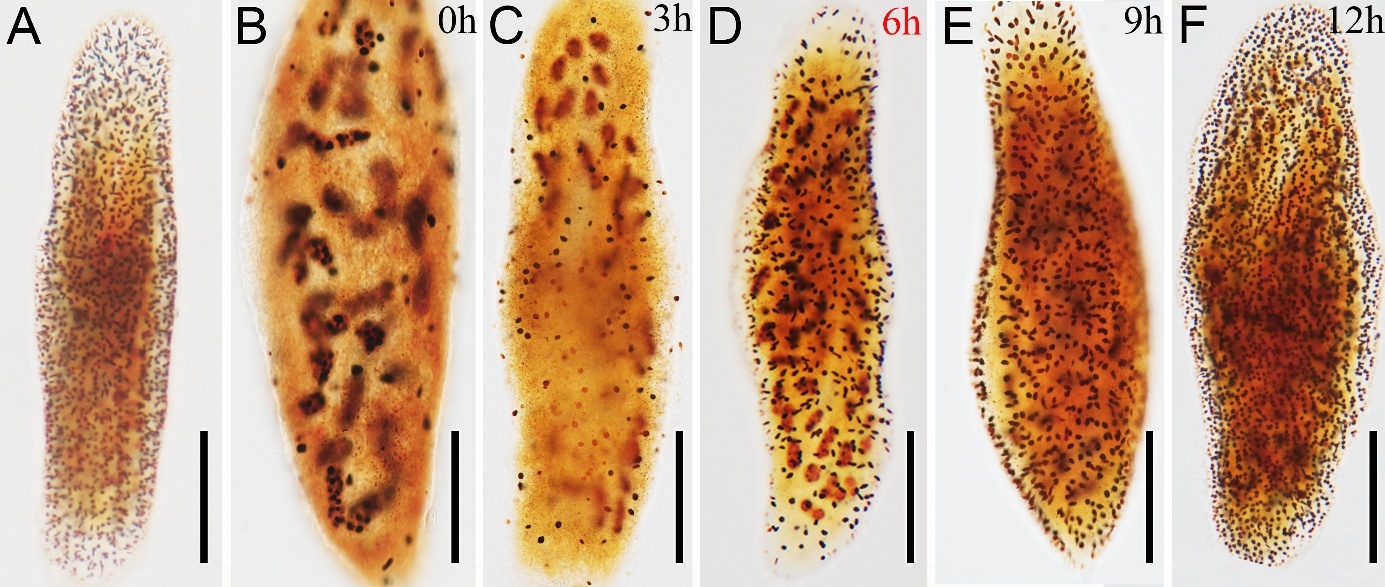


**Fig. S2** Distribution of protrichocysts in *Pseudourostyla cristata* cells after protargol staining. **(A)** Distribution of protrichocysts during the vegetative stage. **(B–E)** Monitored protrichocyst recovery at 3-hour intervals post-treatment (0h, 3 h, 6 h, 9 h, and 12 h post-treatment) using protargol staining. The 6-hour post-treatment time point, labeled in red, represents the regeneration stage and has been chosen for a subsequent transcriptional analysis of regeneration-associated gene expression. Scale bars = 50 μm.

**
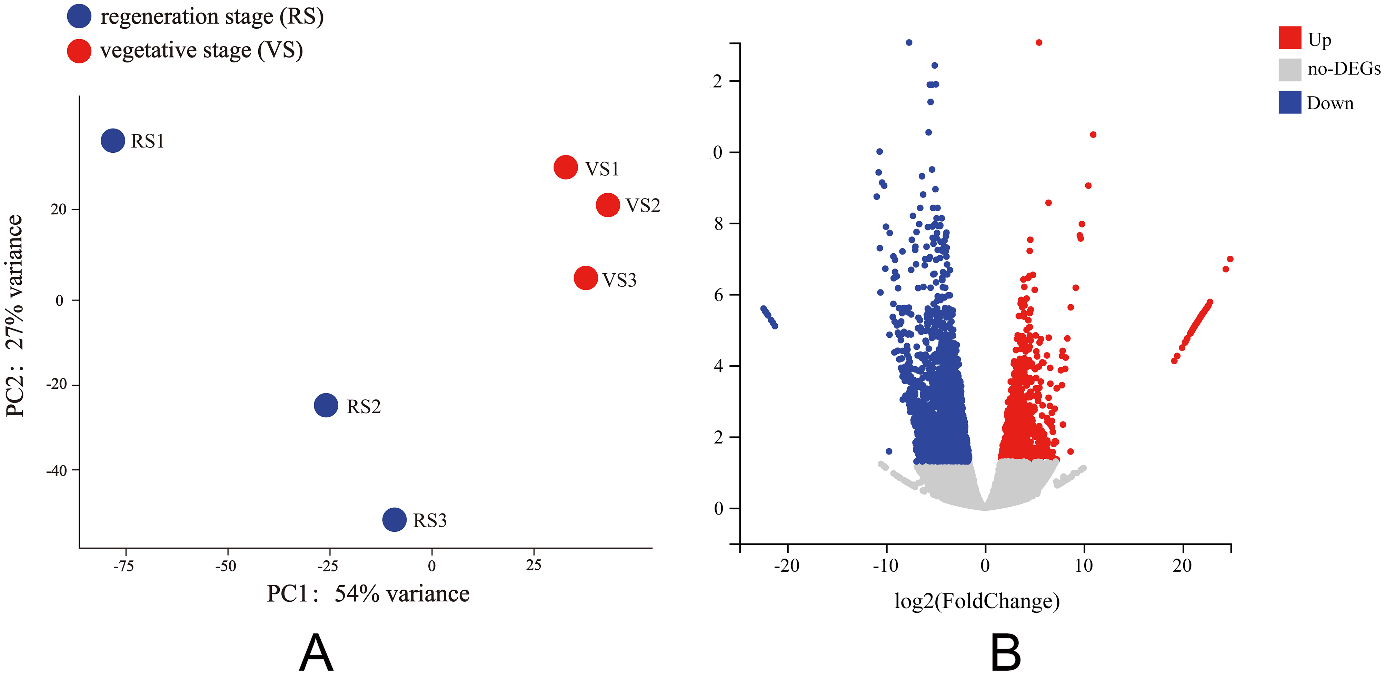
**

**Fig. S3** Transcriptomic profiling of *Pseudourostyla cristata* during protrichocyst regeneration. **A.** Principal component analysis of transcriptomes from regeneration and vegetative stages with three biological replicates each. **B.** Differentially expressed genes (DEGs) between regeneration stage (RS) and vegetative stage (VS). Down, downregulated genes; no-DEGs, non-differentially expressed

genes; Up, upregulated genes.


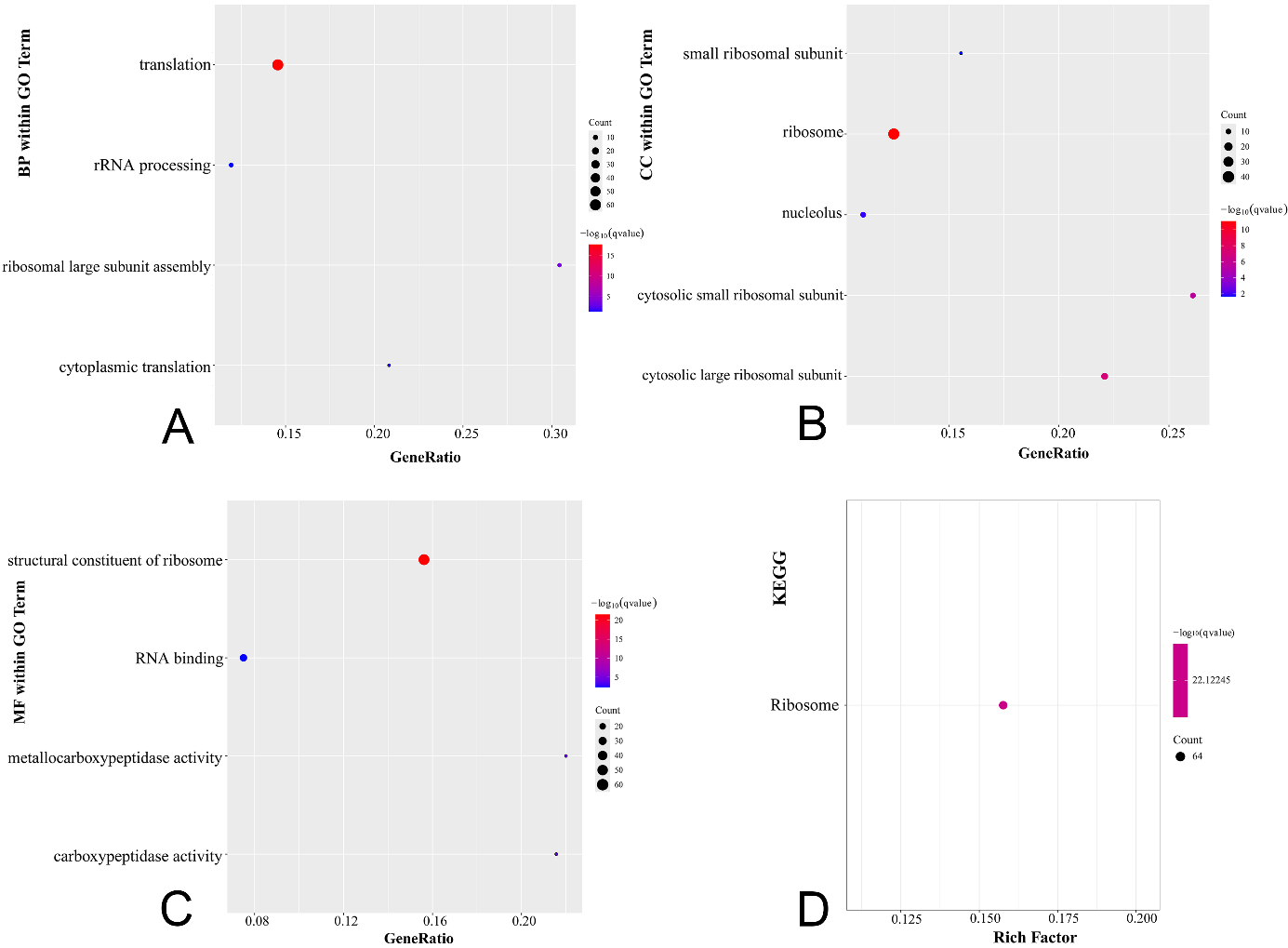


**Fig. S4** Functional **(A–C)** and pathway enrichment **(D)** analysis of upregulated unigenes during *Pseudourostyla cristata*’s protrichocyst regeneration (6h post-treatment). GO, Gene Ontology; BP, Biological Process; CC, Cellular Component; MF, Molecular Function; KEGG, Kyoto Encyclopedia of Genes and Genomes.

**Table S1** Gene Ontology (GO) term enrichment analysis of the 294 upregulated unigenes during regeneration stage (p-value<0.1).

| **GO ID** | **Description** | **Count** | **Ontology** |
| --- | --- | --- | --- |
| GO:0000027 | ribosomal large subunit assembly | 7 | biological_process |
| GO:0002181 | cytoplasmic translation | 5 | biological_process |
| GO:0003723 | RNA binding | 30 | molecular_function |
| GO:0003735 | structural constituent of ribosome | 67 | molecular_function |
| GO:0004180 | carboxypeptidase activity | 11 | molecular_function |
| GO:0004181 | metallocarboxypeptidase activity | 11 | molecular_function |
| GO:0005730 | nucleolus | 12 | cellular_component |
| GO:0005840 | ribosome | 47 | cellular_component |
| GO:0006364 | rRNA processing | 8 | biological_process |
| GO:0006412 | translation | 60 | biological_process |
| GO:0015935 | small ribosomal subunit | 7 | cellular_component |
| GO:0022625 | cytosolic large ribosomal subunit | 17 | cellular_component |
| GO:0022627 | cytosolic small ribosomal subunit | 12 | cellular_component |
